# Computational algorithms to predict Gene Ontology annotations

**DOI:** 10.1186/1471-2105-16-S6-S4

**Published:** 2015-04-17

**Authors:** Pietro Pinoli, Davide Chicco, Marco Masseroli

**Affiliations:** 1 Dipartimento di Elettronica Informazione e Bioingegneria, Politecnico di Milano, Milan, Italy; 2 Institute for Genomics and Bioinformatics, University of California Irvine, Irvine, CA, USA

**Keywords:** Truncated Singular Value Decomposition, Probabilistic Latent Semantic Analysis, weighting schemes, gene clustering, Gene Ontology, biomolecular annotations, Receiver Operating Characteristic

## Abstract

**Background:**

Gene function annotations, which are associations between a gene and a term of a controlled vocabulary describing gene functional features, are of paramount importance in modern biology. Datasets of these annotations, such as the ones provided by the Gene Ontology Consortium, are used to design novel biological experiments and interpret their results. Despite their importance, these sources of information have some known issues. They are incomplete, since biological knowledge is far from being definitive and it rapidly evolves, and some erroneous annotations may be present. Since the curation process of novel annotations is a costly procedure, both in economical and time terms, computational tools that can reliably predict likely annotations, and thus quicken the discovery of new gene annotations, are very useful.

**Methods:**

We used a set of computational algorithms and weighting schemes to infer novel gene annotations from a set of known ones. We used the latent semantic analysis approach, implementing two popular algorithms (Latent Semantic Indexing and Probabilistic Latent Semantic Analysis) and propose a novel method, the Semantic IMproved Latent Semantic Analysis, which adds a clustering step on the set of considered genes. Furthermore, we propose the improvement of these algorithms by weighting the annotations in the input set.

**Results:**

We tested our methods and their weighted variants on the Gene Ontology annotation sets of three model organism genes (*Bos taurus, Danio rerio *and *Drosophila melanogaster *). The methods showed their ability in predicting novel gene annotations and the weighting procedures demonstrated to lead to a valuable improvement, although the obtained results vary according to the dimension of the input annotation set and the considered algorithm.

**Conclusions:**

Out of the three considered methods, the Semantic IMproved Latent Semantic Analysis is the one that provides better results. In particular, when coupled with a proper weighting policy, it is able to predict a significant number of novel annotations, demonstrating to actually be a helpful tool in supporting scientists in the curation process of gene functional annotations.

## Background

In bioinformatics, a *gene function *controlled *annotation *is a key concept; it is the association between a gene (identified by its ID) and a controlled term (identified by its ID and belonging to a terminology or ontology) that describes a specific functional feature. Thus, a controlled gene function annotation states that the involved gene owns the feature (function) described by the considered controlled term. Controlled feature terms are usually included into controlled vocabularies or terminologies, each devoted to a specific aspect (e.g. molecular functions, metabolic features, etc.), and often they are related to other terms of the same vocabulary to form an ontology. In this paper, we consider the feature terms of the Gene Ontology (GO) [1], the well known bioinformatics initiative whose aim is to uniquely and precisely define the features of genes and gene products in a species independent manner. The GO is composed of three controlled vocabularies structured as (almost) separate sub-ontologies: *Biological Process *(BP), which defines a recognized series of molecular events, with a defined beginning and end, pertinent to the functioning of integrated living units (e.g. cells, tissues, organs and organisms); *Cellular Component *(CC), which describes, at the levels of sub-cellular structures and macromolecular complexes, the parts of a cell or its extracellular environment where molecular events occur; and *Molecular Function *(MF), which characterizes the elemental activities of a gene product at the molecular level, such as binding or catalysis. Each GO sub-ontology is structured as a Directed Acyclic Graph (DAG), where every node represents a term (i.e. a concept describing a functional feature) and every edge represents a relation between two concepts, which is mainly of sub-typing ("*is a*") or partition ("*part o*f"). Every sub-ontology tree has a root term, which has the sub-ontology name (BP, CC, MF). In April 2014, the GO contained about 38,600 current terms, describing gene (and gene product) features, with more than 25,550 BP, 9,650 MF and 3,350 CC terms.

Beyond the indubitable importance of ontologies like GO and of gene (and gene product) annotations, they are incomplete and may contain incorrect items. In fact, on one hand, several gene and gene product functions of many organisms have still to be discovered and annotated; on the other hand, many biomolecular annotations are only available as computationally inferred, without the supervision of a human curator. Furthermore, gene and gene product annotations are available in different data banks, maintained by different organizations, which may contain not completely consistent information. Since *in vitro *biomolecular experiments to validate a gene function are costly and lengthy, computational methods and software able to predict and prioritize new biomolecular annotations, e.g. through machine learning algorithms, are an excellent contribution to the field [2]. The techniques discussed in this paper are in this category.

In the last years, several studies dealt with the scientific issue of predicting highly reliable new gene and gene product functional annotations. King et al. propounded decision trees and Bayesian networks to predict novel gene annotations by learning patterns from available annotation profiles [3]. Tao and colleagues [4] advanced by using a k-nearest neighbour (k-NN) classifier, through which a gene inherits the annotations that are common among its nearest neighbour genes in a gene network. The functional distance between genes, based on the semantic similarity of the GO terms used to annotate them, regulates this inheritance process.

New gene functions can also be inferred by taking advantage of multiple heterogeneous data sources. In [5], Barutcuoglu and colleagues used gene expression levels, obtained in microarray experiments, to train a Support Vector Machine (SVM) classifier for each gene annotation to a GO term, and enforced consistency among predicted gene annotations by means of a Bayesian network projected on the GO structure. Conversely, Raychaudhuri et al. [6] and Perez et al. [7] leveraged textual information by mining the literature and extracting keywords that are then mapped to GO concepts.

More recently, to predict novel GO annotations, Zitnik and colleagues used matrix factorization data fusion techniques [8], whereas Vembu and colleagues used a net-work analysis approach [9]. Lavezzo at al. proposed an interesting method based on a genomic sequencing pipeline [10], while in [11] Wang and colleagues illustrated the use of fuzzy logic and rule-based approaches. Conversely, Kordmahalleh et al. proposed the prediction of gene functions based on a *crowding niching-Adaptive mutation *algorithm, which is related to evolutionary multi-modal optimization [12]. Very recently, Yu and colleagues implemented a *weak-label *learning method able to predict protein functions [13].

All these methods worked very well, but no one of them is actually able to predict *new *missing gene functions from a set of available annotations. Instead, given a set of biomolecular annotations and some additional candidate annotations, the listed methods are able to state if the candidate annotations are supposed to be correct or not. On the contrary, some years ago Khatri and colleagues proposed a method [14], built on truncated Singular Value Decomposition (tSVD), which is able to extrapolate gene functions, that is to suggest new gene function annotations absent from the input dataset.

Starting from their proposed tSVD method [14], first we extended it, based on gene clustering, and proposed the Semantically IMproved tSVD (SIM) [15]. Then, similarly to what Khatri et al. did in [16], we enhanced our method by using different annotation frequency and distribution weighting schemes [17]. We also investigated the use of Probabilistic Latent Semantic Analysis (pLSA) [18], a topic modeling method; in [19], we showed its application to predict gene function annotations and discussed the obtained prediction results. Then, we extended also this pLSA implementation with different weighting schemes [20].

In this paper, we summarize the tSVD, SIM and pLSA methods and their variants, benchmark and comparatively discuss them and the results that they provide on different datasets, and highlight some important remarks on their behavior.

The paper organization is as follows. After this introduction, in the *Methods *section we illustrate the considered algorithms and weighting schemes; then in the *Datasets *section we describe the datasets used to comparatively test these methods. In the *Validation procedures *section we illustrate the procedures defined to benchmark the considered algorithms and their variants, and to evaluate the results that they provide. Finally, in the *Results and discussion *section we illustrate some significant results and comparatively discuss them and the considered algorithms. The *Conclusions *section ends.

## Methods

**Figure 1:**
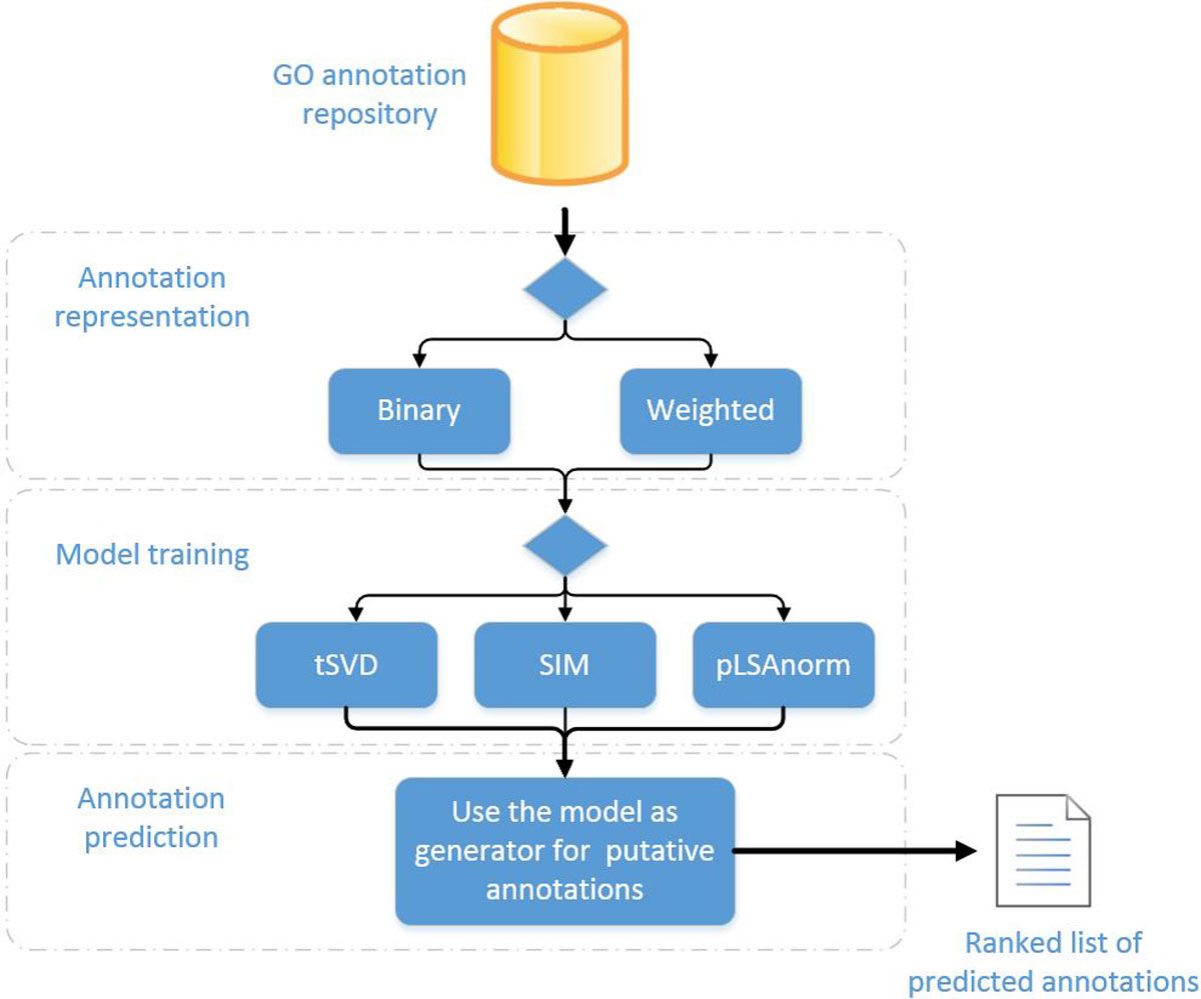
**Prediction workflow**. The input is a gene annotation repository. Firstly, the contained annotations of interest are represented in a computable structure (i.e. a matrix, binary or weighted). Then, this representation is used as training dataset for a machine learning method that fits a predictive model of gene annotations. Finally, the estimated model is treated as a generative process and new putative annotations are produced, along with a confidence value.

With the aim of inferring novel gene annotations, so as to improve the quality and coverage of existing annotation datasets, we implemented the workflow depicted in Figure 1. It is mainly composed of three steps: (a) the set of available annotations is represented in a computable format, then (b) a mathematical model is trained and finally (c) the model is used as a generative process for predicting novel annotations. The output of the workflow is a ranked list of putative associations between genes and function terms, ranked according to a confidence value. For the first and second steps, different variants have been implemented and tested. In the rest of this section we describe the workflow details and variants, as well as the validation procedures that we used to evaluate each workflow variant.

### Binary representation

At the heart of a gene annotation predictive system there is the gene *annotation matrix*. Given an organism, the annotation matrix is built from the set of known annotations of the organism genes to function terms and, in case these terms are related to each other within an ontology, their ontological structure. The ontology structure is essential because, when the controlled terms are organized into an ontology (i.e. along with the terms, also the relationships among them are available, as in the case of the GO), only the gene annotations to the most specific terms describing the gene functional features are directly available; the annotations to the more general terms are implicit in the ontology structure. Thus, in order to build the annotation matrix, we firstly *unfold *the annotation set; this means that, for each annotation of a gene *g *to a term *f *in the annotation set, we add to the set all the annotations between the same gene *g *and each term *f' *such that *f *is a descendant of *f ' *in the ontology structure. We then build a binary matrix **A***_tmp _*of dimensions (*G_tmp _× F_tmp_*), where *G_tmp _*and *F_tmp _*are the number of distinct genes and the number of distinct function terms involved in the unfolded annotations set, respectively. Finally, we apply a further transformation, which we named *pruning*. In this step, we delete from **A***_tmp _*all columns that correspond to terms which are annotated to only few genes, less than *L*, after the annotation unfolding. This step is intended to delete from the input set all those function terms (with their few annotations) that, being very rare, do not bring a significant amount of information about their annotation to distinct genes, but whose presence increases anyway the computational complexity. In the tests following described, in order to improve the computational complexity with practically no impact on the prediction, we heuristically used *L *= 3, since it is very unlikely that the terms annotated only to three genes, out of the several thousands of genes included in each considered dataset, may be predicted annotated to other genes. The reduced matrix returned by the pruning procedure is the annotation matrix **A**. In the following, we use *G *to denote the set of genes whose annotations are represented by the matrix **A**; accordingly, we use *F *to denote the set of function terms in the matrix **A**. Therefore, the annotation matrix **A **is in the [0, 1]*^|G|×|F | ^*space; each of its rows represents a gene, while each of its columns represents a function term. An entry **A**(*g, f *) of the annotation matrix is set to 1 if and only if the gene *g *is annotated either to the term *f *or to any of its descendant terms in the ontology structure (in the case of ontological terms).

We used the annotation matrix as input to the predictive systems designed with the aim of inferring novel unknown gene annotations. The result provided by these systems is a list of associations between a gene and a function term, ranked according to a value that describes how likely the association is; this likelihood is estimated solely based on the available knowledge expressed by the considered annotation set. The predictive methods that we implemented enhance *machine learning *algorithms, such as the *Latent Semantic Indexing *(LSI) by *Singular Value Decomposition *(SVD), *Probabilistic Latent Semantic Analysis*, or weighting schemes such as the *term frequency - inverse document frequency *(TF-IDF) one. These are very general methods used in a broad range of domains; our variants extend them to better fit into the biomolecular annotation domain.

### Weighting the annotation matrix

The annotation matrix that we introduced in the previous section is a simple binary matrix. Studies in the machine learning and information retrieval fields have shown that it is possible to improve the performances of a predictive system, both in term of recall and precision, by moving from the binary matrix to a more complex and informative one. Our approach is to weight the annotation matrix with different weighting schema, exploiting both co-occurrence information and the ontology structure; in our framework, starting from the annotation matrix **A **and the DAG of the term ontology, we build a real valued matrix **W ***∈ *R*^|G|×|F |^*, which becomes the new input of the predictive methods. The weighting schema that we implemented are based on the TF-IDF technique.

The intuitions behind this is that (a) the relevance of a function term for a given gene is proportional to the number of descendant of that terms that are annotated to the gene and (b) if a term is rare (i.e. it is annotated only to a small subset of *G*), it is a better discriminator among the set of genes than common function terms. The former of these two criteria can be captured by the *term frequency *(*TF*) statistics; for each gene *g*, function term *f *and function term *f ^! ^*descendant of *f*, the corresponding term frequency is computed as:

TF(g,f)=1+|{f′:A(g,f′)=1}|

Therefore, for each gene *g *and term *f, T F *(*g, f *) equals either to one, if *g *is directly annotated to *f*, or to one plus the number of descendants of *f *which are associated with *g*, either directly or indirectly, when the annotation of *g *to *f *has been produced by the unfolding process. Thus, general terms with many descendants annotated to a certain gene, are considered more relevant for that gene than terms involved in direct annotations.

The latter criteria can be represented by the *inverse gene frequency *(*IGF *) measure. For each function term *f *it provides an estimation of the importance of an annotation to that term, decreasing the relevance of the annotations to common terms, such as the ones close to the ontology root. Given a function term *f*, we can compute the corresponding *IGF *value as:

$$IGF\left(f\right)=ln\frac{\left|G\right|}{\left|\left\{g:\text{A}\left(g,f\right)=1\right\}\right|}$$

Thus, if a term *f *is associated with all the genes in the corpus, *IGF *(*f *) = *ln*1 = 0; in fact, such term does not provide valuable information, in terms of gene discrimination. On the contrary, if *f *is associated with only one gene, *IGF *(*f *) = *ln|G|*, which is the IGF maximum possible value. In general, if a term is associated with less than $\frac{\left|G\right|}{e}$ genes, the measure *IGF *(*f *) is greater than one and, therefore, its relevance is increased; otherwise, the relevance of the term in the corpus is decreased. We compose this two statistics in order to build distinct weighting schemes; each of such schema is made of three components: (a) a local weight, that represents how much a certain function term is important for a given gene, (b) a global weight, that estimate the relevance of a term in the whole corpus of annotations and (c) a normalization procedure, which is meant to reduce the bias between genes that strongly differ in the number of associated terms.

In Table 1 we report the local and global schemes and the normalization functions that we defined. Each weighting schema is denoted by a three-letter code, the first refers to the local schema, the second to the global one while the last letter refers to the normalization function. Notice that these components are different from the ones proposed by Khatri and colleagues [14] because of the different formulation and meaning of the basic statistics *TF *and *IGF*.

**Table 1:** Weighting schema components.

Code	Name	Description
*Local weight*		

N	No transformation	∀*f, g *: *w_loc _*= *T F *(*g, f *)
A	Augmented	∀*f, g *: *w_loc _*= 0.5 + 0.5 *· *(*T F *(*g, f *)*/max_f _i T F *(*g, f'*))

*Global weight*		

T	Term weight	∀*f *: *w_glob _*= *IGF *(*f *)

*Normalization*		

N	No normalization	Normalization factor is not used
M	Maximum	*wnorm*(*g, f *) = *w*(*g, f *) */ max_f' _w*(*g, f'*)

Each of the proposed weighting schemes is made of a local weight, a global weight and a normalization function. The implemented and tested options for the three components are listed below.

In our previous papers [17] and [20], we tested all possible combinations of the three components of a weighting schema; in this work we consider only the ones that have previously shown better performance (in terms of average improvement with respect to the unweighted case): NTN (*No transformation - Term weight - No normalization*), NTM (*No transformation - Term weight - Maximum*) and ATN (*Augmented - Term weight - No normalization*).

The *weighted annotation matrix ***W **is a (*|G| × |F |*) real-valued matrix; starting from the matrix **A **and a weighting schema, we build such matrix by multiplying each element of **A **by the corresponding local and global weights and (in case) individually normalizing each resulting row (gene profile).

### LSI by truncated singular value decomposition

**Figure 2:**
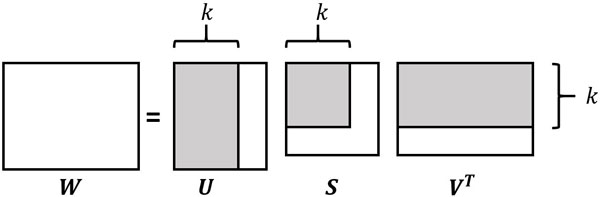
**Truncated Singular Value Decomposition**. Given a truncation level *k*, an approximation of the **W **matrix is built keeping into account only the first *k *columns of the left singular vector matrix **U **and of the right singular vector matrix **V **and the *k × k *portion of the diagonal matrix **S **of the singular values of **W**. Considered sub matrices are highlighted.

The Latent Semantic Indexing [21] technique is build on the tSVD, that is a vectorial latent semantics method. The core of the method is the Singular Value Decomposition (SVD) of the (weighted) annotations matrix. By means of SVD we can rewrite **W **as:

$$\text{W}=\text{US}{\text{V}}^{T}$$

where **U **is an orthonormal (*|G| × p*) real matrix whose columns are the left-singular vectors of **W**, **S **= *diag*(*s*_1_*, s*_2_*, . . ., s_p_*), with *s*_1 _*≥ s*_2 _*≥ . . . ≥ s_p_*, is the (*p × p*) diagonal vector of the sorted singular values of **W**, **V **is an orthonormal (*|F |×p*) real matrix whose columns are the right-singular vectors of **W **and *p *= *min*(*|G|, |F |*). A graphical representation of the SVD matrix decomposition is shown in Figure 2. An interesting property that holds for the matrix **V **is that each of its columns has unitary length; that means that for each column **v***_i_*:

$$length\;\left({\text{v}}_{i}\right)=\sqrt{\overset{\left|F\right|}{\underset{j=1}{\sum }}{\text{v}}_{i}{\left(j\right)}^{2}}=1.$$

Therefore, each of these column vectors defines a direction in the **R***^|F| ^*space, which we can interpret as the space of all the possible annotation profiles of each gene. Each of these directions is then scaled by the related singular value and rotated through the **U **matrix. Thus, the higher the singular value is, the more relevant the corresponding right-singular vector will be.

The tSVD method relies on these considerations and translates them into the following assumption: the directions in the annotation profile space (vector columns of **V**) which correspond to high singular values are likely to represent biologically relevant concepts (seen as relations and co-occurrences of features). In contrast, directions associated with low singular values have high chance to represent *noisy *function term relationships, due both to missing and incorrect annotations in the annotation matrix. The tSVD method discards the direction associated with low singular values; in practices, it builds the approximate matrix:

$$\hat{\text{W}}={\text{U}}_{k}{\text{S}}_{k}{\text{V}}_{k}^{T}$$

where $\hat{\text{W}}$ has the same dimension of the original **W **matrix, **U***_k _*is the matrix made of the first *k *columns of the **U **matrix, **S***_k _*= *diag*(*s*_1_*, . . ., s_k _*) is the diagonal matrix of the first *k *singular values and **V***_k _*is the matrix formed by the first *k *columns of **V**.

For further considerations, it is worth to notice that:

$$\hat{\text{W}}={\text{U}}_{k}{\text{S}}_{k}{\text{V}}_{k}^{T}=\text{W}{\text{V}}_{k}{\text{V}}_{k}^{T}$$

Therefore, given an annotation profile **a **(i.e. a *|F | *sized vector representing the annotations of a given gene), we can compute the approximated annotation vector as:

$$â=\text{a}{\text{V}}_{k}{\text{V}}_{k}^{T}$$

The higher the value of â(f) is, the more confident the method is about the annotation to the feature term *f *of the gene with the profile vector **a**.

### Semantically IMproved tSVD

The tSVD method that we introduced in the previous section is a linear algebra technique; the approximated annotation profiles provided by such method are linear with the computed model. In fact, each computed annotation profile is a linear combination of fixed coefficients, which are the entries of the ${\text{V}}_{k}{\text{V}}_{k}^{T}$ matrix. This property shows a limitation: on average, genes annotated to few terms tend to have a lower predicted annotation value in the  âcomputed annotation profile with respect to genes annotated with a large number of function terms. In fact, let ${\text{V}}_{k}{\text{V}}_{k}^{T}\left(*,j\right)$ be the *j*-th column of the ${\text{V}}_{k}{\text{V}}_{k}^{T}$ matrix; given a gene annotation profile **a**, for each *j *= 1 *. . . |F | *the value of the *j*-th entry of the predicted annotation profile  â is computed as:

$${â}_{j}=\overset{\left|F\right|}{\underset{i=1}{\sum }}a_{i}{\text{V}}_{k}{\text{V}}_{k}^{T}\left(i,j\right).$$

Thus, if **a **includes only a few annotations (i.e. only a few not 0 values), the value of ${â}_{j}$ tends to be low, and on average lower than the one obtained in the case when many values of **a **are not 0, i.e when **a **includes many annotations. In our tests, this was a clear source of bias when applying the tSVD predictive method to genes with a relevant difference in the number of annotated terms. Because of this behavior, the predictive system using the tSVD approach tends to predict lot of annotations for well annotated genes and only a few for poorly annotated ones.

Our Semantically IMproved tSVD (SIM) method is an attempt to overcome this issue, by adding a gene clustering step and defining a specific model for each cluster, i.e. group of more equally annotated genes. The **V **matrix of the tSVD algorithm implicitly uses the term-to-term correlation matrix **T **= **WW***^T^*; in fact **V **is made of the eigen vectors of such matrix. In SIM we propose an adaptive approach: we cluster genes according to their annotation profile and for every gene cluster we estimate a different correlation matrix **T***_c_*, with *c *= 0, 1, 2*, . . ., C*, where *C *denotes the number of clusters and **T**_0 _= **T**. That is, we choose a number C of clusters and completely discard the columns c of matrix **U **where *c > C*, i.e. *c *= *C *+ 1*, . . . , n*. In fact, each column *u_c _*of the SVD matrix **U **represents a cluster and the value *U *(*i, c*) indicates the membership of gene *i *to the *c_th _*cluster. We use this membership degree to cluster the genes (the rows of matrix **W**). The case C = 0 corresponds to the complete **U **matrix; thus, in this case, SIM = tSVD. Then, for each of those **T***_c _*matrices, the **V***_c _*matrix, whose columns are the eigen vectors of *T_c_*, is computed and its columns are sorted in not increasing order, according to the corresponding eigen value. Finally, each **V***_c _*matrix is truncated, keeping into account only the first *k *columns.

In order to build the **T***_c _*matrices we exploit the gene clustering induced by the SVD, which is based on the gene functional similarity. To this end, we consider the **U **matrix, each of whose columns can be interpreted as a cluster. Considered a vector column **u***_i_*, its *j*-th entry is the degree of membership of the *j *gene in the *i *cluster; each gene, can belong to different clusters with different degrees of membership. The construction of the **T***_c _*matrix proceeds as follows: (a) a diagonal matrix **C***_c _∈ ***R***^|G|×|G| ^*is build; the non-zero elements of **C***_c _*are the entries of the **u***_c _*column vector; (b) a modified gene-to-term matrix is computed as **W***_c _*= **C***_c_***W**; (c) finally the matrix **T***_c _*= **W***^T ^***W***_c _*is generated.

Given an annotation profile **a**, the *C *+ 1 different **V***_c,k _*matrices are used to estimate as many predicted annotation profiles:

$${â}_{c}=aV_{c,k}V_{c,k}^{T}.$$

Among them, the best predicted annotation profile is chosen as the one that minimizes the distance from the original annotation profile, as computed by the *L*2 *− norm*:

$$â=arg\underset{c=0,...,C}{\text{min}}||{â}_{c}-a|{|}_{2}$$

That annotation profile is then considered as the predicted one. The entire procedure is repeated for each annotation profiles **a**.

The introduction of the *arg *min operator breaks the strict linearity of the tSVD approach, reducing the bias due to the different number of annotations that different genes have.

### pLSAnorm

The pLSAnorm algorithm is a statistical method that we introduced in [19]; it consists of a slight modification of the pLSA algorithm [18], where the main difference lays on a final normalization step. Similarly to tSVD and SIM, the aim of this method is to build a latent class model to identify hidden relationships among the set of function terms. With respect to the tSVD and SIM methods, which are built on linear algebra foundations, pLSAnorm uses a probabilistic approach based on Bayesian inference. Therefore, while tSVD and SIM compute a  Ŵ that approximates the input (weighted) annotation matrix **W**, pLSAnorm attempts to estimate the probability of the event *"gene g is annotated to the term f"*, for each gene *g *and function term *f*.

**Figure 3:**
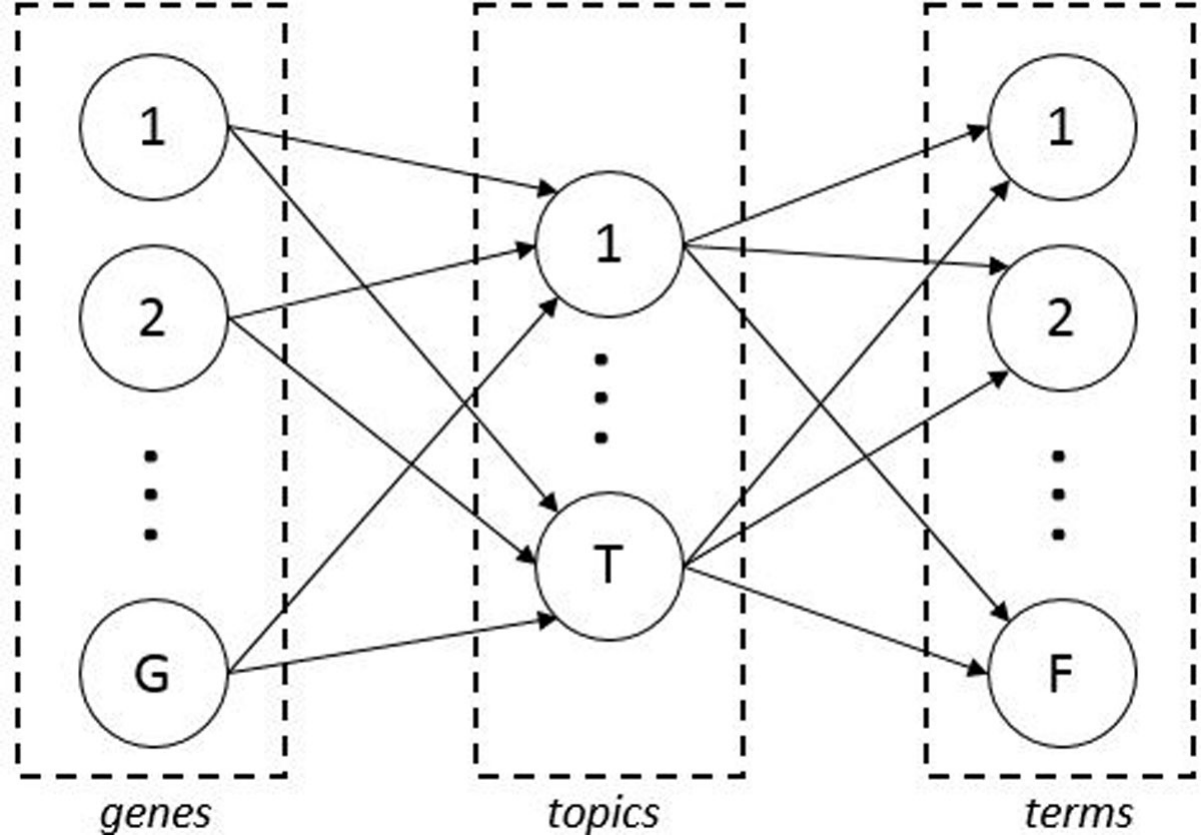
**pLSAnorm aspect model**. Each gene is associated with each function term through hidden variables, the *topics*. Connections between nodes represent probability values.

The core of pLSAnorm is the *aspect model *(depicted in Figure 3), where the latent variables are named *topics *and *T *is the set of all topics. In the aspect model, to each gene *g ∈ G *it corresponds a vector *δ_g _∈ ***R***^|T |^*, for which the property:

$$\underset{t\in T}{\sum }\delta_{g}\left(t\right)=1$$

holds. Thus, we can interpret each of those vectors as multinomial distributions of probabilities over the set of topics, where each element *δ_g_*(*t*) is the probability of having the topic *t *associated with the gene *g*, i.e. *δ_g _*(*t*) = *P *(*t|g*). Similarly, each topic *t ∈ T *corresponds to a vector $\varphi_{t}\in R^{\left|F\right|}$, subject to the constraint:

$$\underset{f\in F}{\sum }\varphi_{t}\left(f\right)=1.$$

In this case, each vector $\varphi_{t}$ can be interpreted as a multinomial probability distribution over the set of function terms and each entry $\varphi_{t}\left(f\right)$ of such vector is the probability of having a function term associated with the topic.

Given the aspect model, the probability of an association between a gene *g *and a function term *f *can be computed as:

(1) $$P\left(f|g\right)=\underset{t\in T}{\sum }\delta_{g}\left(t\right)\varphi_{t}\left(f\right)=\underset{t\in T}{\sum }P\left(f|t\right)P\left(t|g\right).$$

Then, the goal is to estimate from the data all the multinomial distributions such that for each gene and for each term the model produces a valuable estimation of the association probability. The pLSA method estimates the set of all such probability distributions starting from a random initialization and iterating the *Expectation-Maximization *(EM) algorithm. EM estimates a set of value assignments for the probability distributions, which corresponds to a optimum in the likelihood function:

$$ℒ=P\left(\Theta ,\Phi |\text{W}\right)=\underset{g\in G}{\prod }\underset{f\in T}{\prod }{\left(\underset{t\in T}{\sum }P\left(f|t\right)P\left(t|g\right)\right)}^{{\text{W}}_{\left(g,f\right)}}$$

or, equivalently, to a maximum of the log-likelihood function:

$$\ell =\underset{g\in G}{\sum }\underset{f\in F}{\sum }\text{W}\left(g,f\right)\sum \text{log}\left(\underset{t\in T}{\sum }P\left(f|t\right)P\left(t|g\right)\right)$$

where **W**(*g, f *) is the value corresponding to the gene *g *and the function term *t *in the weighted annotation matrix. The EM algorithm is made of two steps: the E-step and the M-step. In the E-step the probability that an association between a gene *g *and a function term *f *is explained by the topic *t *is computed, for each possible combinations of those three entities, by the formula:

$$P\left(t|f,g\right)=\frac{P\left(f|t\right)P\left(t|g,f\right)}{\left|G\right|}$$

In the M-step, the values of Formula 2 are used to estimate a novel value assignment to the probability distributions, which improves the likelihood function value. In details, we compute the new values for the *P *(*t|g*) values as:

$$P\left(t|g\right)=\frac{\sum_{f\in F}W\left(g,f\right)P\left(t|g,f\right)}{\left|G\right|}$$

and the new values for *P*(*f*|*t*)as:

It is important to keep into considerations that the EM algorithms can only find a local optimum of the likelihood function, which may be different from the global one; the reached optimum depends on the random initialization.

Once the assignments for the probability distribution have been computed, it is possible to compute the probability of having an annotation by means of the Formula 1. Notice that, since the vector *P *(*f |g*) has to preserve statistical properties, it is subject to the constraint:

$$\forall_{g}:\underset{f\in F}{\sum }P\left(f|g\right)=1.$$

Again, genes with a high number of suggested annotations will have a lower average prediction value; this introduces some problem in our prediction framework, where we want to rank predictions according to their value. In order to overcome this bias, we introduced a normalization step in which the main difference between pLSAnorm and pLSA lays: for each row of the *P *(*f |g*) matrix we get the maximum *M *and we divide all the entries of that row by *M*, i.e. for each gene we force the function term with the higher probability to have a probability scaled to 1, and all the other term probabilities of the topic are accordingly rescaled. This ensures a better uniformity in the prediction values among different genes. In fact, this mitigates the highlighted issue since after this normalization, for each gene, the terms that are identified as putative annotations have a likelihood value close or equal to one, regardless the total number of predicted annotations for that gene.

### Computational complexity

As reported by Korobkin and colleagues [22], the LSI and pLSA algorithms have the same asymptotic computational complexity; its value is *O*(*N *^2 ^*∗ x ∗ k*), where *N *= *|G| *+ *|F |, x *is the sparsity degree of the annotation matrix used as input to the algorithm and *k *is either the truncation level (in LSI) or the number of topics (in pLSA). The pLSAnorm method we proposed adds a normalization step whose theoretical complexity is *O*(*N*^2^), thus it has the same asymptotic computational complexity that pLSA has. Finally, the complexity of the SIM algorithm can be derived from the LSI one as *O*(*C ∗ N*^2 ^*∗ x ∗ k*), where also the number of clusters *C *is taken into account. As a general indication of the computational time required when these methods are applied on gene annotation data, on the *Danio rerio *dataset (the medium size one of the three that we tested, see following Table 2), the LSI method run for ~5 minutes while both pLSAnorm and SIM took ~20 minutes to complete.

**Table 2:** Quantitative characteristics of the considered GO (BP+CC+MF) gene annotation datasets in their July 2009 version and March 2013 updated version from the GPDW.

Dataset	July 2009			March 2013			#a comparison	
			
	#g	#f	#a	#g	#f	#a	Δ	Δ%
*Curated annotations*								

*Bos taurus*	734	3,714	32,232	2,243	8,421	1,44,358	1,12,126	347.87
*Danio rerio*	1,807	2,967	49,834	3,825	6,848	1,79,142	1,29,308	259.47
*Drosophila m*.	8,722	6,516	3,08,962	10,304	8,850	5,17,457	2,08,495	67.48

*Computational annotations*								

*Bos taurus*	11,646	6,927	3,35,063	5,428	9,107	2,32,945	−102,118	−30.47
*Danio rerio*	14,114	3,270	2,62,940	15,439	4,191	3,45,712	82,772	31.47
*Drosophila m*.	7,950	2,136	86,207	8,433	2,560	96,354	10,147	11.77

Figures do not include GO annotations with IEA or ND evidence, nor obsolete GO terms and genes. #g: number of genes; #f: number of function features (GO terms); #a: number of GO gene annotations; Δ: difference of annotation number between the two dataset versions; Δ%: percentage difference of annotation number between the two dataset versions. *Drosophila m*.: *Drosophila melanogaster *organism.

### Weighting schema influence

In the previous sections we introduced the predictive methods on the **W **matrix; the same methods can be applied without any modification to the **A **matrix, since a binary matrix can always be seen as a real-valued one. On the other hand, the **W **matrix can be seen as the result of the application of a weighting schema to the **A **matrix. By doing so, the weighting schema can modify the results provided by the predictive machine learning method used. In fact, by changing the values of the input matrix, the weighting schemes implicitly change the main directions (i.e. columns) of the **V **matrix computed by the tSVD algorithm; in particular, through the weighting schemes, relevant annotations influence the model selection more than less relevant ones.

The mechanism is very similar also in the SIM method; yet, in this case, the **A **matrix modification by the weighting schemes leads also to a different set of clusters, since also the **U **matrix computed by the tSVD algorithm changes. Generally, such new clusters better reflect the functional similarity of the genes in a cluster. Finally, in the pLSAnorm, changes in the input matrix **A **are translated into a modification of the likelihood function to be maximized; this causes the EM algorithm to end up in a different set of variable assignment for the probability distributions *P *(*f |t*) and *P *(*t|g*) that constitute the predictive model, thus in different annotation prediction likelihoods.

### Input and output matrix comparison

The entries of the reconstructed matrix Ŵ(i,j)>τ are real valued. Given a threshold *τ*, if Ŵ(i,j)>τ, then gene *i *is predicted to be annotated to term *j*. Subject to the original values assumed by the matrix **A**, the following cases may befall:

• If **A**(*i, j*) = 1 and Ŵ(i,j)>τ, the annotation of gene *i *to term *j *is confirmed; this case is denoted as an *annotation confirmed *(AC), with respect to the original **A**(*i, j*). This annotation type can be considered similar to a True Positive (TP).

• If **A**(*i, j*) = 0 and Ŵ(i,j)>τ, a new annotation is suggested; this case is denoted as an *annotation predicted *(AP), with respect to the original **A**(*i, j*). This annotation type can be considered similar to a False Positive (FP). These annotations are those that are inserted in the likely predicted annotation lists generated, which are very useful to biologists and physicians.

• If **A**(*i, j*) = 1 and Ŵ(i,j)≤τ, an existing annotation is suggested to be semantically inconsistent with the available data; this case is denoted as an *annotation to be reviewed *(AR), with respect to the original **A**(*i, j*). This annotation type can be considered similar to a False Negative (FN).

• If **A**(*i, j*) = 0 and Ŵ(i,j)≤τ, the annotation is not present in the original annotation set and it is not suggested by the analysis; this case is denoted as a *not existing annotation confirmed *(NAC), with respect to the original **A**(*i, j*). This annotation type can be considered similar to a True Negative (TN).

We take advantage of these categories to build the Receiver Operating Characteristic (ROC) curves in the validation procedure.

### Datasets

To test the performances of the considered methods, we used three gene annotation datasets obtained from the Genomic and Proteomic Data Warehouse (GPDW) [23,24], a publicly available integrative data warehouse maintained by our group at the Politecnico di Milano [25]. We selected the GO gene annotations of the *Bos taurus *(cattle), *Danio rerio *(zebra fish) and *Drosophila melanogaster *(common fruit fly) organisms. We chose these organisms because their gene annotations to the GO include different and representative numbers of annotations, involved genes and function terms, with curated annotation figures from lower in *Bos taurus *to higher in *Drosophila melanogaster*. We considered two versions of the selected datasets, an older one from July 2009 and an updated one from March 2013. Table 2 provides a quantitative description of all the GO gene annotation datasets considered.

### Validation procedures

The validation of the predicted annotations is a very relevant step in our pipeline. Since we do not have a *gold standard *to refer to, we developed two different validation phases to check the quality of the predictions generated by the different methods considered. The first phase regards the analysis of the Receiver Operating Characteristic curves calculated for each method and their Area Under the Curve (AUC), while the second phase regards the comparison of the predictions to an updated version of the datasets used to determine the predictions.

### Receiver Operating Characteristic curve analysis

A *Receiver Operating Characteristic *(ROC) curve is a graphical plot which depicts the performance of a binary classifier system while its discrimination threshold is varied [26]. Differently from its original definition, we build this curve on *APrate *(on the *x *axis) and *ACrate *(on the *y *axis), where: (see previous section for acronym meaning)

$$APrate=\frac{AP}{AP+NAC}\;and\;ACrate=\frac{AC}{AC+AR}$$

Thus, our ROC curves depict the trade-off between the *APrate *and *ACrate *for all possible values of the threshold *τ *. Notice that, in statistical terms, *APrate *= 1 *− Specificity *and *ACrate *= *Sensitivity*. In our tests, we considered only the *AP rate *in the normalized interval [0, 0.010], in order to evaluate the best predicted annotations (APs) having the highest likelihood score (since the more NACs are present, the closer the *APrate *is to zero).

This ROC curve analysis is an efficient tool to understand the dissimilarity between the input and the output annotations. A ROC curve showing a high *Area Under the Curve *(AUC) corresponds to having many ACs (annotations present in input and confirmed present in output) and many NACs (annotations absent in input and confirmed absent in output). This means that the output matrix is very similar to the input matrix, and the output gene annotation profiles strongly reflect the input ones. On the contrary, a low AUC means a lot of differences between the input and the output annotations. However, difficulties are related to using this ROC curve indicator in our application scenario. In fact, since the output predictions are compared to the input annotations, if the prediction system did not predict any new annotation, the maximum AUC value (100%) would be obtained. This would seem to mean an optimal prediction, but actually it would give no useful information about new annotations. Despite this, we consider the ROC curve analysis a good dissimilarity indicator also in our application scenario, although less useful and precise than other validation methods, such as the one based on the comparison of the prediction results to an updated version of the dataset used to generate the predictions.

### Dataset version comparison

We also implemented an alternative validation procedure, which is based on the comparison of the obtained predictions to an updated version of the dataset used to determine the predictions. In our tests, as input we used sets of GO gene annotations available on July 2009, and as updated datasets the same sets of GO gene annotations available on March 2013.

The first step of this validation procedure consists of building the predictive system model taking into account only the most reliable annotations in the older dataset considered for the prediction, i.e the ones not computationally inferred or with information actually available about the genes or gene products being annotated. Thus, we ignore the GO gene annotations with *Inferred Electronic Annotation *(IEA) or *No biological Data available *(ND) evidence code. Then, we use the created model in order to predict a list of candidate annotations that are not present in the considered dataset with evidence different from IEA or ND. Finally, we count how many of these predicted annotations are present (a) in the considered dataset with computational (IEA or ND) evidence, (b) in the updated dataset with any evidence, or (c) in the updated dataset with not-computational (IEA or ND) evidence. The higher those counts are, the better the predictive system behaves, i.e. it is able to predict an higher number of confirmed annotations that were unknown when the dataset used for the prediction was created. In particular, the count of predicted annotations that are confirmed by annotations with not computational evidence in the dataset updated version is the most relevant and reliable one. In fact, such annotations are reviewed by curators and mostly experimentally confirmed. It is important to notice that all these counts only provide a lower estimate of the prediction precision; predicted annotations that are not found in the dataset updated version can be correct, but not found just simply because they have not been discovered yet by other means.

## Results and discussion

Our validation tests aim at comparing the performances of the three considered methods and their combinations with different weighting schemes. In order to do so, for each method we chose the same number of latent classes (i.e. principal components in the tSVD and SIM methods and topics in the pLSAnorm method); we set them heuristically to 500, as was done in [14], in order to be able to compare our results also to those reported by Khatri and colleagues. Furthermore, for the SIM method we decided to set to 3 the number of clusters to be used; this value was estimated taking into account both computational complexity and goodness of results. We ran several experiments with different numbers of clusters and noticed that, with the considered datasets, usually results did not change much when we used a cluster number greater than 3 (data not shown). Since the greater the number of clusters is, the higher the computational complexity of the annotation prediction is, we chose to always use 3 clusters.

We show the results of the ROC analysis validation in the next subsection and the results of the dataset version comparison analysis in the following section.

### ROC analysis results

**Figure 4:**
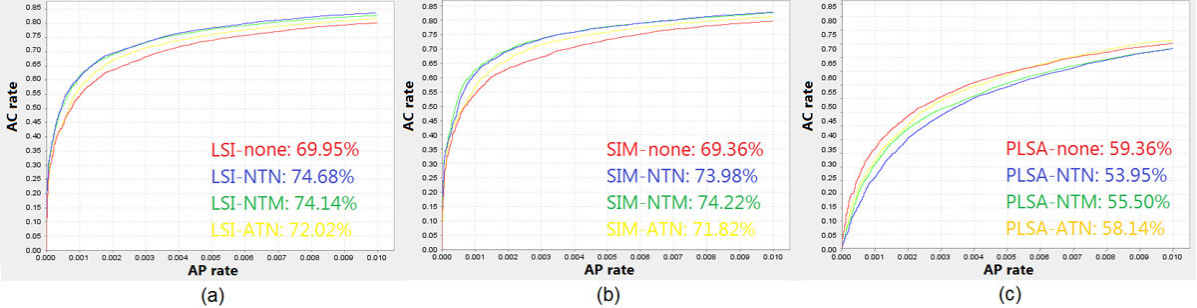
**ROC curves for the Bos taurus datasets**. ROC curves and their AUC percentages of *Annotation Confirmed rate *(AC rate) versus *Annotation Predicted rate *(AP rate), obtained by varying the threshold *τ *in predicting the GO annotations of *Bos taurus *genes with the LSI (a), SIM (b) or pLSA (c) methods, each with or without weighting schemes.

The performed ROC analysis validation provided similar ROC curves and AUC values for the three annotation datasets considered, i.e. the *Bos taurus, Danio rerio *and *Drosophila melanogaster *organism ones. As an example, we show in Figure 4 the ROC curves and AUC percentages of all the methods and their weighting schema variants that we applied to the *Bos taurus *dataset. We can observe that all the LSI and SIM method variants with different weighting schemes provide better results than the pLSA ones. In fact, all the LSI and SIM ROC AUCs are greater than an indicative threshold of 66.66% (equal to the 2/3 of the possible maximum AUC value), which represents limitedly acceptable values; whereas all the pLSA method variants with the different weighting schemes considered show ROC AUC values less than such threshold. Furthermore, in the LSI and SIM methods we can also observe that the NTN and NTM schemes outperform the other ATN and no schema variants, showing the highest AUC percentages. On the contrary, the prediction quality of the pLSA method worsens when the weighting schemes are added. In fact, in Figure 4 one might notice that the pLSA method with no weighting schema shows the highest AUC percentage (59.36%) among all the pLSA method variants. ROC curves and AUC values obtained for the *Danio rerio *and *Drosophila melanogaster *datasets show similar trends.

**Figure 5:**
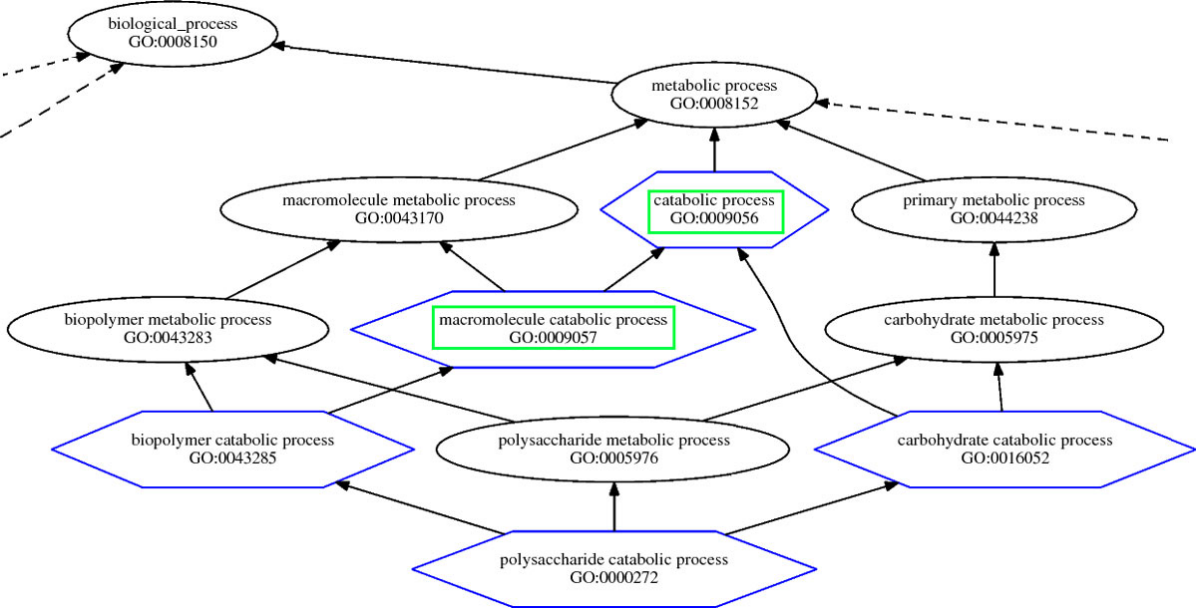
**Predictions for the PGRP-LB gene**. Branch of the Directed Acyclic Graph of the GO Biological Process terms associated with the *PGRP-LB Peptidoglycan recognition protein LB *gene (Entrez Gene ID: 41379) of the *Drosophila melanogaster *organism. It includes GO terms present in the analyzed dataset (black circles), as well as GO terms predicted by the SIM method with the NTM weighting schema as associated with the same gene (blue hexagons) and the ones of them that were found validated in the dataset updated version (green rectangles). Other GO DAG parts are connected to the shown branch as indicated by the dotted lines.

As an example of our gene annotation predictions, we report in Figure 5 a branch of the Directed Acyclic Graph of the GO Biological Process terms predicted by the SIM method, with the NTM weighting schema, as associated with the *PGRP-LB Peptidoglycan recognition protein LB *gene (Entrez Gene ID: 41379) of the *Drosophila melanogaster *organism. One may notice that, in this sub-tree, our SIM method predicted five new annotations, in addition to the six that were already present. Out of these five predicted annotations, two (*catabolic process *- GO:0009056 and *macromolecole catabolic process *- GO:0009057) were found validated with reliable evidence in the used dataset updated version. These confirmations suggest the likely correctness of their direct children, *biopolymer catabolic process *- GO:0043285 and *carbohydrate catabolic process *- GO:0016052, both also children of terms annotated to the same gene with reliable evidence in the dataset used for the prediction.

### Dataset version comparison results

In Table 3 we report the validation results obtained by comparing the annotations predicted by each considered method and its weighting schema variants to the updated version of the annotation datasets used to generate the predictions. For each dataset, every prediction method returns a list of predicted annotations sorted according to their likelihood value. We considered the top 500 annotations of each list and evaluated the percentages of such annotations that were found confirmed by a) annotations present in the dataset used for the prediction, but with computational evidence only (*cmp*), i.e. not considered to perform the predictions, b) any annotation in the updated version of the considered dataset (*Uany *), or c) curated annotations in the updated version of the considered dataset (*Ucur*), i.e. with a not-computational evidence. The latter values (*Ucur*) are the most relevant and important, since they refer to predicted annotations with supervised biological confirmation. In Table 3, at a first sight, they could seem low, but it is important to notice that they represent only the predicted annotations found most reliably confirmed after 44 months; many other predicted annotations (even all) may be correct, but not found confirmed in the dataset updated version just because at the time of its creation they were not discovered yet.

**Table 3:** Percentages of the top 500 predicted annotations found confirmed for each method (LSI, SIM, pLSA), weighting schema (none, NTN, NTM, ATN), and dataset (*Bos taurus, Danio rerio, Drosophila melanogaster*).

	*Bos Taurus*			*Danio rerio*			*Drosophila m*.		
**Method**	**cmp**	**Uany**	**Ucur**	**cmp**	**Uany**	**Ucur**	**cmp**	**Uany**	**Ucur**

LSI-none	**26.4**	26	3.6	**13**	21.2	11.6	**31.2**	**30**	6.8

LSI-NTN	22.2	24.8	7.2	9.4	27.4	16.8	6.6	13.8	7.2

LSI-NTM	14.8	19.2	6.4	6.6	17.6	11.6	9.8	26	**17**

LSI-ATN	21.6	**28.2**	**9.4**	6.2	**27.6**	**22.6**	23.6	24	5.8

SIM-none	19.2	19	4.4	**12.4**	21.8	10.8	**32.6**	35	10.6

SIM-NTN	17.4	20.6	7	11.4	28.8	17.8	7.4	24.6	17

SIM-NTM	22	24	6.2	7.4	30.2	21.4	16	**45.6**	**35.2**

SIM-ATN	**24**	**32.2**	**10.4**	6	**31.6**	**26.8**	22.6	23.8	6

pLSA-none	**27.4**	20.6	5.2	14	**24.4**	13	4.8	6.6	4.2

pLSA-NTN	19.6	**21.2**	**6.8**	11.8	21.6	**13.2**	**8**	**11.6**	**5.6**

pLSA-NTM	14.6	20.2	5	**15.8**	23.8	11.6	5.4	7	3.2

pLSA-ATN	15.6	16.2	6.4	4.8	9.6	5	3.8	6.4	4

We report: the portion of predictions that were confirmed in the outdated dataset with only computational evidence, therefore not included in the input corpus (*cmp*); the portion of predictions that were confirmed in the updated dataset with any evidence (*Uany*) and the portion of confirmed predictions in the updated dataset with not-computational evidence (*Ucur*). The best values obtained for each of these three cases and dataset across the considered methods are highlighted in bold.

Inspecting the figures in Table 3, we can see that when the three methods are run without any weighting schema, they provide similar results, even if pLSA gives predictions significantly worst than the other two methods on the *Drosophila melanogaster *dataset, i.e. in the case of a very high number of available annotations considered for the prediction (see Table 2). (Please note that only available curated annotations were considered for the prediction). Furthermore, the weighting schemes generally provide benefits to the predictive methods and some interesting trends emerge from the results. pLSA performances are always improved by the NTN schema, in particular for to the predictions validated by the reliable curated annotations in the more recent version of the dataset. Conversely, pLSA performances are usually decreased by the other weighting schemes. With the LSI and SIM methods, the ATN schema provides good improvements when small datasets are analyzed; with the growth of the dataset size, the NTM schema becomes the best one for those methods. Interestingly, the values in Table 3 show that, in general, weighting schemes lead to a better prediction system, since they seem to avoid predicting those annotations that, in the updated version of the annotation set considered, are present only with computational evidence, i.e. are less likely to be biologically correct. In fact, according to the figures in Table 3 related to the number of predictions confirmed with biological evidence (Ucur), any method applied to the binary annotation representation is always outperformed by at least one of its weighting schema enhanced variants.

Finally, overall the SIM algorithm that we proposed, coupled with a proper weighting schema, provides the best results with respect to the other considered methods, also when they are enhanced with any weighting schema. This improvement is particularly evident when keeping into account the predicted annotations confirmed by annotations with curated evidence in the dataset updated version (*Ucur*). In contrast, the pLSA method is the worst of the three methods compared; such outcome may depend on the overfitting issues that are known to affect pLSA, which are amplified in a context where the training samples are both incomplete and partially incorrect, as in the annotation datasets.

## Conclusions

In this paper we discussed and comparatively evaluated three computational methods to predict novel gene (or gene product) functional annotations from a set of known ones. For each method, we implemented and tested four different variants, obtained by applying on each method three distinct weighting schemes. Performance evaluations were performed on three distinct annotation datasets of different sizes, i.e. *Bos taurus, Danio rerio *and *Drosophila melanogaster *gene annotations to GO terms. Obtained results show that our proposed SIM method is a valuable tool for gene annotation prediction and biological hypotheses design. Comparisons indicate SIM as the most precise method of the considered ones, in contrast to the pLSAnorm method which shows limitations in this gene annotation prediction application. Furthermore, the proposed annotation weighting schemes lead to significant prediction improvements, although different specific schemes provide better results for different sizes of the evaluated dataset and predictive method used.

By leveraging the lists of the most likely biomolecular annotations that our computational algorithms can predict, scientists might be able to address their research in a more focused direction, possibly avoiding time-consuming and expensive biomolecular experiments for gene function determinations. The main application and goal of our work is to *suggest *some gene functions that are more likely to exist to scientists, who can consider them in designing and prioritizing their experiments.

Our future work will address advantages and issues in taking into account also other gene (and gene product) annotation types, such as the ones regarding pathways (e.g. from KEGG [27], or Reactome [28]), or diseases (e.g. from OMIM [29], or GAD [30]). We plan also to implement and test other *topic modeling *methods, such as Latent Dirichlet Allocation (LDA) [31], as well as new on-line machine learning techniques, such as Hybrid stochastic-adversarial on-line learning [32]. We also aim at using new prioritization techniques able to reveal most likely predicted annotations through their ontology tree structure [33]. On the validation side, we intend to take advantage of literature-based validation software, as made in [34], and implement useful statistical coefficient for ROC analysis, as made by Robin et al. [35]. Finally, we want to integrate our software into the on-line Web platform of Bio Search Computing [36] and make it publicly available to the scientific community.

## List of used abbreviations

AC: Annotation Confirmed

AP: Annotation Predicted

AR: Annotation to be Reviewed

ATM: Augmented - Term weight - Maximum

AUC: Area Under the Curve

BP: Biological Process

CC: Cellular Component

cmp: Computational evidence

DAG: Directed Acyclic Graph

FN: False Negative

FP: False Positive

GAD: Genetic Association Database

GO: Gene Ontology

GPDW: Genomic and Proteomic Data Warehouse

ID: Identifier

IDF: Inverse Document Frequency

IEA: Inferred from Electronic Annotation

IGF: Inverse Gene Frequency

k-NN: k-Nearest Neighbour

KEGG: Kyoto Encyclopedia of Genes and Genomes

LDA: Latent Dirichlet Allocation

LSI: Latent Semantic Indexing

MF: Molecular Function

NAC: No Annotation Confirmed

ND: No biological Data available

NTM: No transformation - Term weight - Maximum

NTN: No transformation - Term weight - No normalization

O: Order of

OMIM: Online Mendelian Inheritance in Man

PGRP-LB: Peptidoglycan recognition protein LB

pLSA: Probablistic Latent Semantic Analysis

PPME1: Protein phosphatase methylesterase-1

ROC: Receiver Operating Characteristic

SIM: Semantically Improved tSVD

SVD: Singular Value Decomposition

SVM: Support Vector Machine

TF: Term Frequency

TN: True Negative

TP: True Positive

tSVD: Truncated Singular Value Decomposition

Uany: Any evidence in Updated dataset

Ucur: Curated (not computational) evidence in Updated dataset

## Competing interests

The authors declare that they have no competing interests.

## Authors' contributions

PP designed and implemented the pLSAnorm algorithm and the variants of the LSI and SIM algorithms enhanced with the weighting schemes, run the dataset comparison tests and contributed to write this article.

DC run the ROC tests and contributed to write this article.

MM conceived the original strategy of the project, supervised the project tasks and contributed to write this article.

## Declarations

Publication costs were covered by "Data-Driven Genomic Computing (GenData 2020)" PRIN project (2013-2015), funded by the Italy's Ministry of the University and Research (MIUR).

This article has been published as part of *BMC Bioinformatics *Volume 16 Supplement 6, 2015: Selected articles from the 10th International Meeting on Computational Intelligence Methods for Bioinformatics and Biostatistics. The full contents of the supplement are available online at http://www.biomedcentral.com/bmcbioinformatics/supplements/16/S6.
